# Prevalence and Clinical Features of FOG in Chinese PD Patients, a Multicenter and Cross-Sectional Clinical Study

**DOI:** 10.3389/fneur.2021.568841

**Published:** 2021-03-08

**Authors:** Jing Gan, Weiguo Liu, Xuebing Cao, Anmu Xie, Wentao Li, Canxing Yuan, Lirong Jin, Suzhi Liu, Lingjing Jin, Dengjun Guo, Yuefei Shen, Yuncheng Wu, Zhenguo Liu

**Affiliations:** ^1^Department of Neurology, School of Medicine, Xinhua Hospital Affiliated to Shanghai Jiaotong University, Shanghai, China; ^2^Department of Neurology, Nanjing Brain Hospital Affiliated Nanjing Medical University, Nanjing, China; ^3^Department of Neurology, Union Hospital, Tongji Medical College, Huazhong University of Science and Technology, Wuhan, China; ^4^Department of Neurology, The Affiliated Hospital of Qingdao University, Qingdao, China; ^5^Department of Neurology, Shanghai Municipal Hospital of Traditional Chinese Medicine, Shanghai University of Traditional Chinese Medicine, Shanghai, China; ^6^Department of Neurology, Longhua Hospital, Shanghai University of Traditional Chinese Medicine, Shanghai, China; ^7^Department of Neurology, Zhongshan Hospital, Fudan University, Shanghai, China; ^8^Department of Neurology, The Affiliated Taizhou Hospital, Wenzhou Medical University, Taizhou, China; ^9^Department of Neurology, Shanghai Tongji Hospital, Tongji University School of Medicine, Shanghai, China; ^10^Department of Neurology, Tongde Hospital of Zhejiang Province, Hangzhou, China; ^11^Department of Neurology, The First Affiliated Hospital of Guangxi Medical University, Nanning, China; ^12^Department of Neurology, Shanghai General Hospital, Shanghai Jiao Tong University School of Medicine, Shanghai, China

**Keywords:** parkinson's disease (PD), freezing of gait (FOG), anxiety, quality of life, prevalence, epidemiological investigation

## Abstract

**Objectives:** Freezing of gait (FOG) is generally considered as an independent symptom of Parkinson's disease (PD) with a complex pathophysiology. There is a wide range of associated clinical features of FOG reported from different studies without consistent conclusion. Thus, a multicenter, cross-sectional study was designed to investigate the prevalence and clinical features of FOG together with its unique contribution quality of life in Chinese PD patients.

**Methods:** Eight hundred and thirty eight PD patients were consecutively recruited into this study from 12 hospital centers in six provinces in China. Clinical information, including motor and neuropsychological features as well as pharmacological details, was collected.

**Results:** Of 827 PD patients, 245 (29.63%) reported FOG. The prevalence of FOG was strongly correlated with modified H-Y stages and symptomatic duration (*p* < 0.01). 84.90% freezers experienced FOG during turning and 88.98% experienced when initiating the first step. Compared with non-freezers, freezers reported longer disease duration (7.73 ± 5.44 vs. 4.69 ± 3.94, *p* < 0.000), higher frequent PIGD phenotype (61.22 vs. 35.91%, *p* < 0.000), higher scores of UPDRS III (32.85 ± 15.47 vs. 22.38 ± 12.89, *p* < 0.000), HAMA (10.99 ± 7.41 vs. 7.59 ± 6.47, *p* < 0.000), HAMD (15.29 ± 10.29 vs. 10.58 ± 8.97, *p* < 0.000) and lower MMSE score (25.12 ± 5.27 vs. 26.63 ± 3.97, *p* < 0.000), and higher daily levodopa dosage (432.65 ± 264.31 vs. 319.19 ± 229.15, *p* < 0.000) with less frequent initial use of dopaminergic agonist (8.57 vs. 14.78%, *p* < 0.05). Using binary logistic regression, the associated factors of FOG might be non-tremor dominant onset (OR = 3.817, *p* < 0.000), the presence of anxiety (OR = 2.048, *p* < 0.000) and imbalance (OR = 4.320, *p* = 0.012). Freezers had poorer quality of life than non-freezers and FOG impacted PDQ-8 independently.

**Conclusion:** Nearly one third of the PD patients experienced FOG. Its frequency increased with PD progression and FOG reduced independently the quality of life. Non-tremor dominant, disease progression, and anxiety were risk factors of FOG.

## Introduction

Freezing of gait (FOG) was one of the most disabling symptoms in Parkinson's disease (PD). FOG was defined as “a brief episodic absence or marked reduction of forward progression of the feet despite the intention to walk” (1). Its sudden and unpredictable nature contributed to PD patients' fallings, which lead to the immobility and loss of independence (1, 2). Currently, the pathogenesis of FOG is still unclear. Studies demonstrated that FOG was poorly associated with parkinsonian cardinal motor features and had selective response to levodopa (1, 3). It suggested that non-dopaminergic pathophysiologic mechanisms might be involved in FOG development. As a poor indicator of PD patients' quality of life (QoL), FOG still lacks effective treatments. Therefore, it would be more meaningful to clarify the risk factors of FOG to help clinicians make proper treatment therapies to delay the occurrence of FOG.

The prevalence and clinical factors related to FOG have been reported in several studies (4–16). However, the data varied depending on diverse detection methods or genetic backgrounds or populations (1, 4–13). It reached no consistent conclusion on the FOG associated factors of gender, motor fluctuation, some non-motor symptoms like hallucination, depression, anxiety as well as the usage of antimuscarinic drugs (6–12, 14–16). Notably, the relationship between dopamine replacement therapy and FOG was debated (5, 6, 12, 14), and little information was acquired about FOG's association with the initial antiparkinsonian medications which was an interventive factor. Additionally, PD patients always experienced various motor and non-motor symptoms, it was unclear about FOG' contribution value to the decline of PD-QoL among all these symptoms.

At this background, we conducted this large sample, multi-center, cross-sectional study in Chinese PD patients to clarify the FOG's prevalence and associated factors, together with its unique contribution to the QoL.

## Patients and Methods

### Participants

PD patients' data were obtained from a multicenter, cross-sectional, observational study (clinical registration No: NCT03026595). The recruitment, which lasted for 1 year from October 2017 to November 2018, was conducted in outpatient clinics and hospitalization in 12 hospital centers from six different regions of China. All participants were diagnosed with PD according to the UK Brain Bank Diagnostic Criteria for PD, with the age above 18 years old. The exclusion criteria were as follows: (1) who had atypical or secondary parkinsonism, (2) who were pregnant or lactating, (3) who were unable to cooperate with the assessment, (4) who had participated other clinical research within the recent 30 days. All patients involved in this study gave their signed informed consent form. The study was approved by the Research Ethics Committee of each center of each study center.

### Data Collection

Before the recruitment, all neurologists were trained for clinical assessments for this study together to reduce the bias. Standard demographic details, including age, gender, disease duration, onset age, and onset site, were collected. Patients were evaluated at their “ON” status. The clinical assessments included the Unified Parkinson's Disease Rating Scale (UPDRS), modified Hoehn and Yahr scale, Berg Balance Scales (BBS), the Mini-Mental Scale Examination (MMSE), Hamilton Anxiety Rating Scale (HAMA), Hamilton Depression Rating Scale (HAMD) (24 items), and the 8-item Parkinson's Disease Questionnaire (PDQ-8). PD motor subtype was classified as tremor dominant (TD), posture instability and gait difficulties (PIGD), or intermediate (IND), based on the UPDRS scores (7). According to BBS, cumulative points above 40 are considered as “normal,” a range of 21–40 points considered as “decreased balance ability” implying that patients could walk with assistance, points below 20 (inclusive) considered as “poor balance” implying that patients need to use wheelchairs (17). Patients would be considered as having anxiety or depression if they had eight points or more of HAMA or HAMD scales, respectively (18). Cognitive dysfunction was defined as MMSE <24 points for alphabets or <17 points for analphabets (19, 20). Information of pharmacological treatment was collected in detail, and levodopa daily dose (LDD) and levodopa equivalent daily dose (LEDD) were finally accounted (21).

FOG was assessed via a self-report structured questionnaire named the new FOG questionnaire (NFOG-Q) which was developed by Nieuwboer et al. (22). It included three parts. Part I was used to detect FOG (patients and/or their caregivers recall if patients feel/present feet get glued to the floor while walking, making a turn, or start walking during the past month). Part II was used to assess the severity of FOG. Part III was used to assess the impact of FOG on daily life.

### Statistical Analysis

All data were presented as the percentage or mean ± standard deviations (SD). Descriptive statistics received normality test. Bivariate analysis was performed with Student's *t*-test or **χ2** test as appropriate between PD patients with and without FOG. Analysis of variance was used to evaluate the relation between levodopa dosage and freezing span. A binary logistic regression analysis based on forward stepwise method was conducted to determine the significant variables which were correlated with FOG. The presence of FOG or not was used as a dependent variable. The variables with significant differences in univariate analysis were used as covariables, including disease duration, onset age, PD subtypes, onset lower limbs or not, H-Y stages, motor fluctuation or not, dyskinesia or not, balance stages, DA agonist as initial treatment or not, anxiety or not, depressive or not, cognitive impairment or not, LDD, and LEDD. The results were shown with odds ratio (OR) and 95% confidence intervals (CIs). Spearman correlation analysis was used to determine the major influencing factors of PDQ-8. Hierarchical regression analysis was used to determine the FOG's impact contributing value to the quality of life. Quality of life (PDQ-8) was used as a dependent variable. The major influencing factors of PDQ-8 were used as covariables. These variables were divided into three groups: group I was the scores of mood and cognition (HAMD, HAMA, and MMSE scores); group II, the scores of motor function (UPDRS III and IV scores); and group III was the score of FOG. Then, we put the variables of group I into the first level and variables of group II into the second level during the hierarchical regression analysis based on a stepwise method. Controlling the influencing factors (mood disorder, cognitive impairment, and motor dysfunction) which were also associated with PDQ-8, the independent impact value of FOG on quality of life was analyzed. The multicollinearity was absent for the model. Statistical Package for the Social Science (SPSS) version 25 was used in the analysis. A significance level of 0.05 was set for all statistical tests.

## Results

### FOG Point Prevalence and Distribution in Chinese PD Study Population

Eight hundred and thirty eight PD patients were screened in this study, of them, 3 patients were excluded for incooperation, 1 patient was excluded because of participating other clinical research, 6 patients were excluded from the analysis due to the missing data on NFOG-Q score. Finally, 827 PD patients were included into analysis. According to the NFOG-Q part I, 245 patients reported FOG, and the point prevalence of FOG was 29.63%. The mean score of NFOG-Q part II was 11.36 ± 4.64 (0–19) and that of NFOG-Q part III was 4.96±2.11 (0–9).

**Figure 1:**
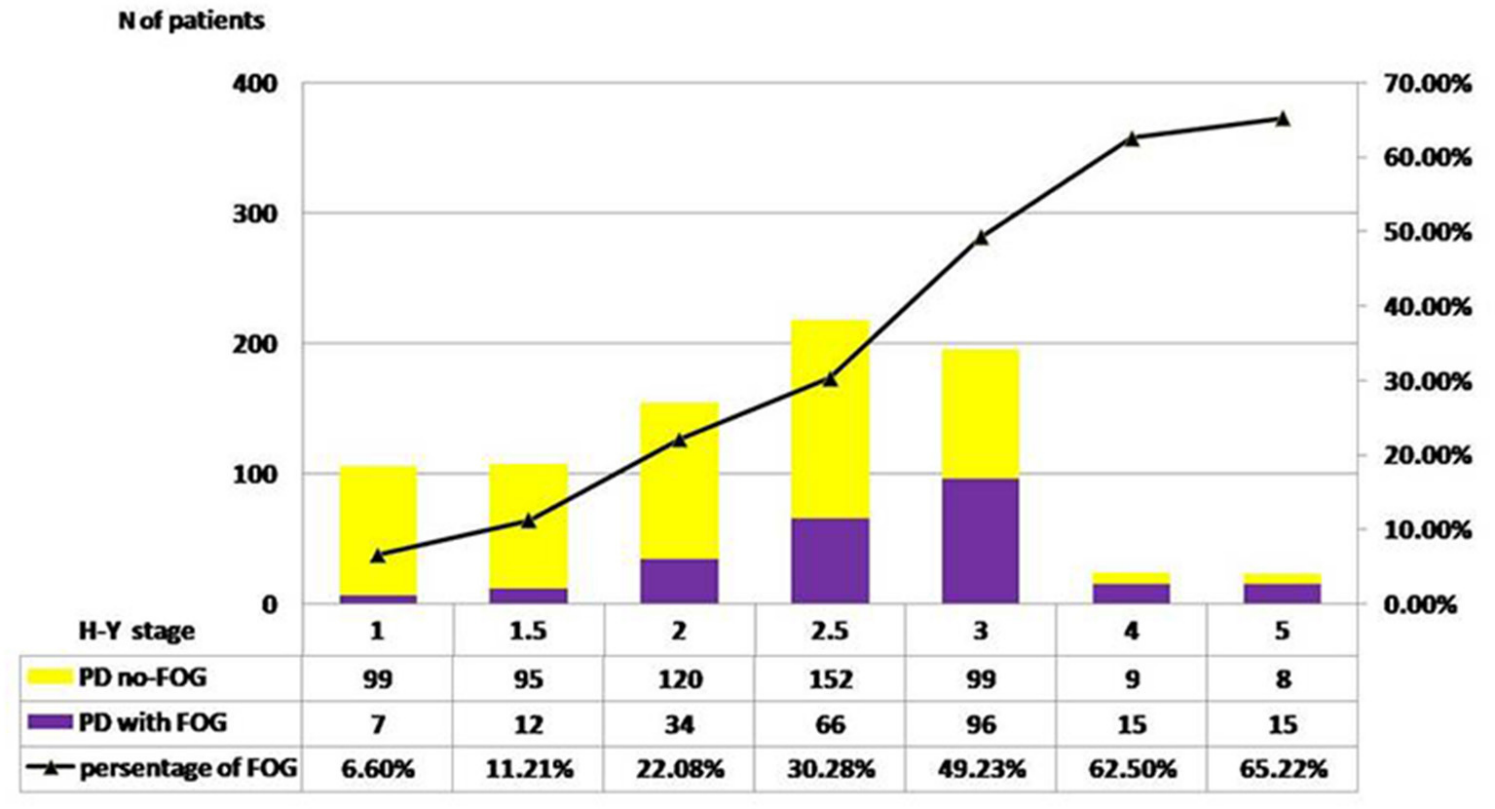
Pareto chart of FOG frequency across H-Y stages. H-Y 1.0, the prevalence of FOG was 6.60%;H-Y 1.5, the prevalence was 11.21%; H-Y 2.0, 22.08%; H-Y 2.5, 30.28%; H-Y 3.0, 49.23%; H-Y 4.0, 62.50%; H-Y 5.0, 65.22%. A significant linear trend across H-Y stages was disclosed by result χ^2^ test (*p* < 0.01).

**Figure 2:**
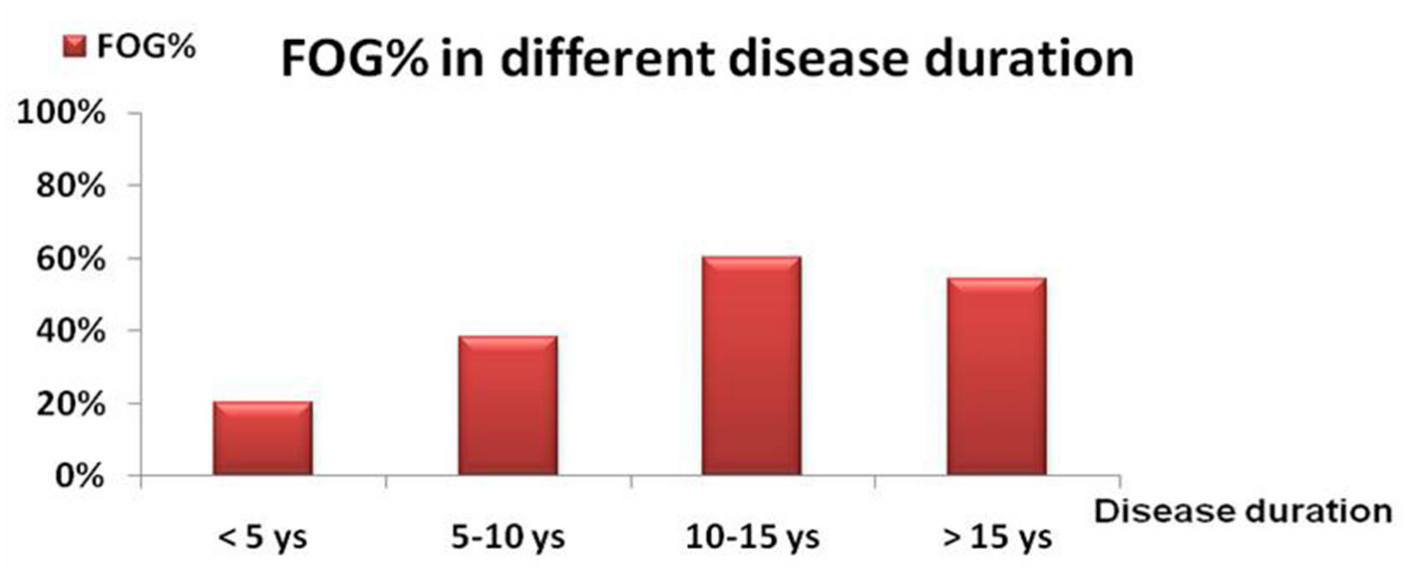
The frequency of FOG (%) in different symptomatic durations. The frequency of FOG was 20% in PD patients with clinical course of <5 years; 38.42% in PD patients with a course with 5–10 years; 60.27% in PD patients with a course with 10–15 years and 54.0% in PD patient with a course with > 15years.

The distribution of FOG in different H-Y stages was shown in Figure 1. The prevalence of FOG at the stage of H-Y 1.0 was 6.60% and gradually increased to the highest value of 65.22% at H-Y 5.0. The prevalence of FOG was statistically different among different H-Y stages (*p* < 0.01). Moreover, the frequency of FOG increased with the progression of the disease duration. FOG was identified in 20.0% of PD patients with disease duration <5 years, 38.42% in disease duration between 5 and 10 years, 60.27% in disease duration between 10 and 15 years, and 54.0% in disease duration over 15 years (Table 1 and Figure 2).

**Table 1:** Demographic details of PD patients with or without FOG in this study.

	**PD patients with FOG (*n* = 245)**	**PD patients without FOG (*n* = 582)**	**t /χ2 value**	***p***
Age (mean ± SD)^a^	65.76 ± 9.30	65.27 ± 8.91	−0.709	0.478
Gender, male(%)^b^	57.55%	52.23%	1.961	> 0.05
H-Y stage (mean ± SD)^a^	2.78 ± 0.83	2.11 ± 0.79	0.215	0.000
Disease duration (mean ± SD)^a^	7.73 ± 5.44	4.69 ± 3.94	−7.896	0.000
<5 years (%)^b^	42.21%	70.74%	73.363	<0.01
5–10 years (%)	31.97%	21.51%		
10–15 years (%)	18.03%	4.99%		
> 15 years (%)	7.79%	2.75%		
Onset age (mean ± SD)^a^	58.10 ± 10.50	60.58 ± 9.24	3.194	0.002
<50 years (%)^b^	23.67%	14.60%	9.638	<0.01
≥ 50 years (%)	76.33%	85.40%		
PD subtypes^b^				
TD (%)	22.45%	56.36%	80.649	<0.01
PIGD (%)	61.22%	35.91%		
IND (%)	16.33%	7.73%		
Onset symptoms^b^				
Onset side (left, %)	47.45%	48.85%	0.111	> 0.05
Onset site (lower limbs, %)	44.81%	32.46%	9.063	<0.01
UPDRS Total scores (mean ± SD)^a^	57.26 ± 22.34	36.74 ± 19.20	−12.548	0.000
UPDRS I	4.11 ± 2.50	2.77 ± 2.27	−7.521	0.000
UPDRS II	16.64 ± 6.88	9.89 ± 5.38	−13.690	0.000
UPDRS III	32.85 ± 15.47	22.38 ± 12.89	−9.315	0.000
UPDRS IV	3.26 ± 3.04	1.71 ± 2.82	−6.862	0.000
PD motor fluctuations (%)^b^	44.49%	17.70%	65.569	<0.01
Dyskinesia (%)^b^	23.67%	4.81%	65.838	<0.01
Berg balance scores (mean ± SD)^a^	42.11 ± 12.66	49.94 ± 7.79	8.988	0.000
Antiparkinsonian medication^b^				
LD+DA (%)	56.33%	48.97%	3.735	> 0.05
Levodopa monotherapy (%)	15.51%	22.85%	5.667	<0.05
DA agonist monotherapy (%)	2.04%	8.59%	10.884	<0.01
MAOIs (%)	23.27%	14.60%	9.093	<0.01
Levodopa as initial treatment (%)	57.60%	56.70%	0.186	> 0.05
DA agonist as initial treatment (%)	8.57%	14.78%	6.094	<0.05
LDD/day (mean ± SD)	432.65 ± 264.31	319.19 ± 229.15	−5.834	0.000
LEDD/day (mean ± SD)	514.90 ± 303.70	382.92 ± 242.11	−6.020	0.000
HAMA (mean ± SD)^a^	10.99 ± 7.41	7.59 ± 6.47	−6.230	0.000
HAMD (mean ± SD)^a^	15.29 ± 10.29	10.58 ± 8.97	−6.219	0.000
MMSE (mean ± SD)^a^	25.12 ± 5.27	26.63 ± 3.97	3.840	0.000
PDQ-8 (mean ± SD)^a^	8.56 ± 5.33	5.29 ± 5.18	−8.088	0.000

a *Data were performed for group differences with Student's t-test*.

b *Data were performed for group differences with Chi-square test*.

Ninety three PD patients with FOG (93/245, 37.96%) experienced very frequent freezing events (frequency > once a day), 65 patients (26.53%) experienced freezing once a day, 60 (24.49%) had freezing once a week, and 27 (11.02%) had occasional freezing (< once a week). Mostly, FOG occurred at the episodes of turning (208/245, 84.90%) or initiating the first step (218 cases, 88.98%). The time span of freezing usually lasted for 5–30 s in nearly half of the patients with turning FOG, more than 30 s in 29% of patients with turning FOG. In the contrast, for the FOG that occurred in patients initiating the first step, the freezing span lasted for 2–5 s in 50.67% of patients with FOG and 5–30 s in 23.11% in patients with FOG.

### Clinical Features of FOG in Chinese PD Patients

Demographic, clinical characteristics, and medical information of the study populations were detailed in Table 1.

### Clinical Characteristics Difference Between FOG and Non-FOG Patients

Compared with the non-FOG patients, FOG patients had a higher percentage of lower limb onset as the initial motor symptoms (44.81 vs. 32.46%, *p* < 0.01), PIGD motor types (61.22 vs. 35.91%, *p* < 0.01), early-onset PD (onset age <50 yrs) (23.67 vs. 14.60%, *p* < 0.01), and a younger onset age (58.10 ± 10.50 vs. 60.58 ± 9.24, *p* = 0.002).

In terms of motor symptoms, FOG patients had higher UPDRS III (32.85 ± 15.47 vs. 22.38 ± 12.89, *p* < 0.000) and UPDRS IV scores (3.26 ± 3.04 vs. 1.71 ± 2.82, *p* < 0.000), higher H-Y stage (2.78 ± 0.83 vs. 2.11 ± 0.79, *p* < 0.000), and higher percentage of motor complications than non-FOG patients. Freezers had lower balance score than that of non-freezers (42.11 ± 12.66 vs. 49.94 ± 7.79, *p* < 0.000). With respect to neuropsychological symptoms, FOG patients had higher scores of UPDRS I (4.11 ± 2.50 vs. 2.77 ± 2.27, *p* < 0.000), HAMA (10.99 ± 7.41 vs. 7.59 ± 6.47, *p* < 0.000), and HAMD (15.29 ± 10.29 vs. 10.58 ± 8.97, *p* < 0.000) whereas a lower MMSE score (25.12 ± 5.27 vs. 26.63 ± 3.97, *p* < 0.000), compared with non-FOG patients.

In addition, FOG patients received higher dosage of LDD and LEDD than non-FOG patients. In terms of the medication types, we found FOG patients were less frequently administrated with dopamine receptor agonist (DA) as the initial anti-PD medication treatment (8.57% vs. 14.78%, *p* < 0.05), however, there was no significant difference in initial levedopa use between two groups. Moreover, FOG patients were treated more frequently with combination medications, compared to non-FOG patients. The use frequency of monoamine oxidase B inhibitors (MAOIs) was much higher in FOG patients than in non-FOG patients (23.27 vs. 14.60%, *p* < 0.01). The One-way ANOVA indicated that “the freezing episode span in turning” was related neither to LDD nor to LEDD (*F* = 1.541, *p* = 0.191 and *F* = 1.085, *p* = 0.364, respectively). Similarly, no correlation was found between “the freezing episode span in initiating the first step” and LDD or LEDD (*F* = 1.034, *p* = 0.391 and *F* = 1.069, *p* = 0.372, respectively).

FOG patients experienced worse quality of life than non-freezers according to PDQ-8 (8.56 ± 5.33 vs. 5.29 ± 5.18, *p* < 0.000) and UPDRS II (16.64 ± 6.88 vs. 9.89 ± 5.38, *p* < 0.000).

### Clinical Influencing Factors of FOG

Variables with significantly statistical difference between FOG and non-FOG groups were further included into the binary logistic regression analysis, including disease durations, onset age, PD subtypes (tremor or non-tremor), onset lower limbs or not, H-Y stages, motor fluctuation or not, dyskinesia or not, balance stages, DA agonist as initial treatment or not, anxiety or not, depression or not, cognitive impairment or not, LDD, and LEDD. Finally, freezing was associated with younger onset age (OR = 0.973, 95% CI = 0.953–0.995, *p* = 0.015), longer disease duration (OR = 1.058, 95% CI = 1.011–1.108, *p* = 0.015), non-tremor phenotype onset (OR = 3.817, 95% CI = 2.550–5.714, *p* < 0.000), advanced H-Y stage (OR = 1.621, 95% CI = 1.238–2.124, *p* < 0.000), worse balance (OR = 4.320, 95% CI = 1.381–13.517, *p* = 0.012), the presence of motor complications and anxiety (OR = 2.048, 95% CI = 1.407–2.983, *p* < 0.000). (shown in Table 2).

**Table 2:** Clinical factors related to FOG in this study.

**Variables**		**OR (95% CI)**	***p***
Disease duration		1.058 (1.011–1.108)	0.015
Onset age		0.973 (0.953–0.995)	0.015
Phenotype	Tremor	1.00	0.000
	No-tremor	3.817 (2.550–5.714)	
H-Y stage		1.621 (1.238–2.124)	0.000
Presence of dyskinesia		2.339 (1.280–4.274)	0.006
Presence of motor fluctuation		2.035 (1.335–3.102)	0.001
Balance stage	Normal	1.00	
	Decreased balance ability	4.320 (1.381–13.517)	0.012
	Poor balance	1.964 (1.138–3.391)	0.015
HAMA scores ≥ 8		2.048 (1.407–2.983)	0.000

*A logistic regression analysis based on forward stepwise method was conducted to determine the most significant variables which were correlated with FOG in PD*.

### Relationship Between FOG and the Quality of Life

The relationship between FOG and the quality of life was analyzed in PD patients with FOG. PDQ-8 score was positively correlated with the scores of UPDRS III (*r* = 0.346, *p* < 0.000), NFOG (*r* = 0.324, *p* < 0.000), UPDRS IV (*r* = 0.298, *p* < 0.000), HAMD (*r* = 0.657, *p* < 0.000), HAMA (*r* = 0.616, *p* < 0.000), and negatively correlated with the scores of MMSE (*r* = −0.237, *p* < 0.000) and BBS (*r* = −0.423, *p* < 0.000). PDQ-8 score was not associated with the onset age (*r* = −0.012, *p* = 0.852) or sex distribution (*r* = −0.012, *p* = 0.846). This indicated that parkinsonians' quality of life was associated with motor symptoms (including FOG), motor complications, mood, and cognitive impairment.

Then, a hierarchical regression analysis was used to determine the FOG's contributing value to the quality of life. Controlling the influencing factors (mood disorder, cognitive impairment, and motor dysfunction), we analyzed the impact of FOG on quality of life independently. Finally, the analysis indicated that the scores of HAMD, UPDRS III, and FOG had the strongest impact on the PDQ-8 scores, with the contributing value of 54.1% among all included variables. The FOG score was an independent influencing factor of PDQ-8, with the contributing value of 2.3% (shown in Table 3).

**Table 3:** A hierarchical regression model of the PDQ-8 scale.

	**Adjusted *R*^**2**^**	***R*^**2**^ change**	**Standardized beta**	***t***	***p***
Step 1	0.462	-	-	-	0.000
Step 2	0.520	0.062	-	-	0.000
Step 3	0.541	0.023	-	-	0.001
HAMD score	-	-	0.544	10.812	0.000
UPDRS III score	-	-	0.193	3.805	0.000
FOG impact score	-	-	0.169	3.300	0.001

*The hierarchical regression was used to determine the impact of FOG on quality of life of PD patients. The significant variables were divided into three groups: group I: the HAMD, HAMA scores and the MMSE score, group II: the UPDRS III score, UPDRS IV score, group III: FOG score. Then, the variables of group I were put into the first level and variables of group II into the second level during the hierarchical regression analysis based on stepwise method. Controlling the factors of group I and II, the independent contributing value of FOG on quality of life was 2.3%. The multicollinearity was absent for the final model. The Tolerance of HAMD, MMSE, HAMA, UPDRS III, UPDRS IV and FOG was 0.832, 0.840, 0.282, 0.817, 0.898 and 0.803, respectively. The VIF was 1.201, 1.191, 3.548, 1.224, 1.113, and 1.248, respectively*.

## Discussion

In this study, we found that FOG increasingly occurred during the progression of PD and played an independent negative impact on patients' quality of life. Moreover, PD patients with non-tremor phenotype or anxiety were more likely to develop FOG. The patients with non-tremor phenotype would be 3.5 times more likely to occur FOG than tremor phenotype.

The prevalence of FOG ranged from 7% of *de novo* PD patients (4) to 81% of PD patients with disease duration over 20-years (8–11, 23) from the literatures. In our study, the FOG point prevalence was nearly one third (29.63%) in mixed patients with early and advanced phase who were still ambulatory. Our prevalence was quite lower than the other results (7, 11). It might be attributed to a higher percentage (71.6%) of PD patients at a lower H-Y stage (1–2.5). In addition, the difference in the inclusion criteria and genetic backgrounds may contribute to the discrepancy of the prevalence among studies. Our data confirmed that FOG patients had a longer disease duration and were at a more advanced disease stage, experienced a higher incidence of motor complications and poorer balance, a greater amount of dopaminergic therapy as well as worse quality of life, which was consistent with previous reports (1, 6, 7, 9–11, 14, 24). These results strongly supported that FOG was associated with PD progression (25).

Our data showed that the percentage of PIGD motor phenotype was almost double as high as non-FOG patients. The binary regression analysis showed that non-tremor motor phenotype was a powerful potential influencing factor of FOG. Previous studies also found that (11, 24, 26) PD-FOG patients tended to be dominated by PIGD motor phenotype. This suggested different pathologic progression underlaid the two motor phenotypes of PD. The postmortem findings indicated that patients initially presenting with a tremor-dominant motor phenotype had a more serious limbic Lewy bodies burden whereas patients with a non-tremor-dominant motor phenotypes had a more serious neocortical Lewy bodies burden (24, 27). In our study, the frequency of lower limbs onset was much higher in the FOG group than that in the non-FOG group, but it was not associated with FOG in the final logistic analysis. Currently, the association between the lower limbs of onset symptom and FOG controversial (8, 12, 26, 28), which still required further long-term follow-up to confirm.

There was little information about the association between initial antiparkinsonian medications and FOG. We wanted to know whether different medication types in the initiation of anti-parkinsonian treatment would have a different impact on the occurrence of FOG. Of all the medication types, we found that the non-FOG group used more DA as initial medical treatment than the FOG group, however, the regression analysis failed to show that a close relation between the first drug type choice and FOG. Our initial anti-PD drugs were collected retrospectively, there might be recall bias. Initial antiparkinsonian treatments were first analyzed in two groups of freezers and non-freezers. Previous follow-up studies were rare and did not focus on the correlation between initial antiparkinsonian medications and FOG occurrence (25, 27). Although longer levodopa treatment duration was found to be associated with FOG (1), the mechanism was still unknown. It was not clear what role levodopa play in the development of FOG (29, 30). Alternatively, given that dopaminergic dysfunction might impact the degree to which network loops cross over, dopamine agonists may play a crucial role in the development of FOG (26). Thus, the relation between dopaminergic medicaments and FOG may be worth exploring in future research.

Our results confirmed that FOG patients have a more frequent and serious neuropsychological symptoms, which was consistent with previous studies (1, 24–26, 31). It has been suggested that neuropsychological symptoms were not only related to FOG but also involved in the pathophysiology of this phenomenon (1, 15, 29, 31, 32). In our study, of these neuropsychological symptoms, only anxiety was found as a risk factor for FOG onset in the logistic regression model. Our patients with anxiety were twice as likely to present FOG as those without anxiety. Many evidences supported that anxiety closely correlated with FOG. Studies indicated that there were high levels of anxiety in PD patients with FOG and anxiety could predict the onset of FOG at 1-year follow up (15, 25–31). A recent longitudinal study indicated that anxiety was a strong predictor of FOG with the accuracy of 82.1% that predicting FOG development in the next 15 months (26). Anxiety contributed not only to the frequent occurrence of FOG but also to a longer duration of FOG episodes (33). The cerebral network hypothesis that involved in the interaction between gait and emotion was confirmed by functional imaging studies, which found an increased striato-limbic connectivity as well as a lack of top-down control by frontal-parietal network over amygdale in FOG PD patients. Dysfunctional limbic circuitry was involved in the pathogenesis of FOG (15, 30, 31). These theories implied the relation between anxiety and FOG. Depression and cognitive impairment failed to become clinical predictors of FOG statistically in our study, although there were different results and opinions (1, 8, 24, 28). Thus, the long-term follow-up studies were necessary for us to conduct the relationship between neuropsychological symptoms and FOG.

Finally, we confirmed that FOG was an independent negative influencing factor of quality of life (34). The contribution of FOG impact score to the model was 2.3% uniquely while controlling for other factors which were also associated with PDQ. The impact of FOG was the third largest of all factors, only next to depression and motor severity.

These results gave us some clinical implications. Although the progress of PD cannot be stopped presently, we can carry out disease modification treatment in early stage or optimize the pharmacological treatment to delay PD progression. For no-tremor PD patients, gait and balance should be regularly assessed and early gait rehabilitation training. Early detection of anxiety and early intervention may play a role in delaying the occurrence FOG. Our study has several limitations. First, we did not differentiate the “ON” or “OFF” status of FOG. This information might be useful to explore the association between dopamine therapy and FOG (24). Secondly, we did not evaluate frontal function, which was reported to be closely related to FOG occurrence (6). Future research on FOG should include all frontal lobe-related symptoms. Thirdly, this was a cross-sectional study and the relation between FOG and the clinical variables still required a longitudinal evaluation to be testified.

## Conclusion

Our epidemiology study showed that the prevalence of FOG in China was nearly one third in our ambulatory PD patients and was strongly associated with disease progression. Freezers experienced poor quality of life independently influenced by FOG. No-tremor phenotype, disease progression, and presence of anxiety were significant FOG risk factors. Future prospective study should be done to determine accurate clinical predictors and further exploring the mechanism of FOG.

## Data Availability Statement

The datasets generated for this study are available on request to the corresponding author.

## Ethics Statement

The studies involving human participants were reviewed and approved by Ethical approval for the original study was obtained from the Ethics Committee of Xinhua Hospital affiliated to Shanghai Jiaotong University School of Medicine. The patients/participants provided their written informed consent to participate in this study.

## Author Contributions

JG and ZL were involved in the manuscript preparation, writing of the first draft, and statistical analysis with design and execution. WLiu, XC, AX, WLi, CY, LirJ, SL, LinJ, DG, YS, and YW were involved in the initiation of the project, organization, and execution of the project. All authors read and approved the final manuscript.

## Conflict of Interest

The authors declare that the research was conducted in the absence of any commercial or financial relationships that could be construed as a potential conflict of interest.

## References

[1] Nutt JG Bloem BR Giladi N Hallett M Horak FB Nieuwboer A. Freezing of gait: moving forward on a mysterious clinical phenomenon. Lancet Neurol. (2011) 10:734–44. 10.1016/S1474-4422(11)70143-0 21777828 PMC7293393

[2] Okuma Y Silva de Lima AL Fukae J Bloem BR Snijders AH. A prospective study of falls in relation to freezing of gait and response fluctuations in Parkinson's disease. Parkinsonism Relat Disord. (2018) 46:30–5. 10.1016/j.parkreldis.2017.10.013 29079421

[3] Bartels AL Balash Y Gurevich T Schaafsma JD Hausdorff JM Giladi N. Relationship between freezing of gait (FOG) and other features of Parkinson's: FOG is not correlated with bradykinesia. J Clin Neurosci. (2003) 10:584–8. 10.1016/S0967-5868(03)00192-9 12948464

[4] Giladi N McDermott MP Fahn S Przedborski S Jankovic J Stern M. Freezing of gait in PD: prospective assessment in the DATATOP cohort. Neurology. (2001) 56:1712–21. 10.1212/WNL.56.12.1712 11425939

[5] Ge HL Chen XY Lin YX Ge TJ Yu LH Lin ZY. The prevalence of freezing of gait in Parkinson's disease and in patients with different disease durations and severities. Chin Neurosurg J. (2020) 6:17. 10.1186/s41016-020-00197-y 32922946 PMC7398304

[6] Perez-Lloret S Negre-Pages L Damier P Delval A Derkinderen P Destée A. Prevalence, determinants, and effect on quality of life of freezing of gait in Parkinson disease. JAMA Neurol. (2014) 71:884–90. 10.1001/jamaneurol.2014.753 24839938

[7] Amboni M Stocchi F Abbruzzese G Morgante L Onofrj M Ruggieri S. Prevalence and associated features of self-reported freezing of gait in Parkinson disease: The DEEP FOG study. Parkinsonism Relat Disord. (2015) 21:644–9. 10.1016/j.parkreldis.2015.03.028 25899545

[8] Ou R Guo X Song W Cao B Yang J Wei Q. Freezing of gait in Chinese patients with Parkinson disease. J Neurol Sci. (2014) 345:56–60. 10.1016/j.jns.2014.07.002 25043665

[9] Sawada M Wada-Isoe K Hanajima R Nakashima K. Clinical features of freezing of gait in Parkinson's disease patients. Brain Behav. (2019) 9:e01244. 10.1002/brb3.1244 30851088 PMC6456785

[10] Hall JM Shine JM O'Callaghan C Walton CC Gilat M Naismith SL. Freezing of Gait and its associations in the early and advanced clinical motor stages of parkinson's disease: a cross-sectional study. J Parkinsons Dis. (2015) 5:881–91. 10.3233/JPD-150581 26444088

[11] Macht M Kaussner Y Möller JC Stiasny-Kolster K Eggert KM Krüger HP. Predictors of freezing in Parkinson's disease: a survey of 6,620 patients. Mov Disord. (2007) 22:953–56. 10.1002/mds.21458 17377927

[12] Ou R Wei Q Cao B Song W Hou Y Liu H. Predictors of freezing of gait in Chinese patients with Parkinson's disease. Brain Behav. (2018) 8:e00931. 10.1002/brb3.931 29541542 PMC5840456

[13] Tang X Yu L Yang J Guo W Liu Y Xu Y. Association of sleep disturbance and freezing of gait in Parkinson disease: prevention/delay implications. J Clin Sleep Med. (2020). 10.5664/jcsm.9022. [Epub ahead of print]. 33231167 PMC8020700

[14] Zhang H Yin X Ouyang Z Chen J Zhou S Zhang C. A prospective study of freezing of gait with early Parkinson disease in Chinese patients. Medicine. (2016) 95:e4056. 10.1097/MD.0000000000004056 27368041 PMC4937955

[15] Avanzino L Lagravinese G Abbruzzese G Pelosin E. Relationships between gait and emotion in Parkinson's disease: a narrative review. Gait Posture. (2018) 65:57–64. 10.1016/j.gaitpost.2018.06.171 30558947

[16] Banks SJ Bayram E Shan G LaBelle DR Bluett B. Non-motor predictors of freezing of gait in Parkinson's disease. Gait Posture. (2019) 68:311–6. 10.1016/j.gaitpost.2018.12.009 30553992 PMC6773271

[17] Downs S Marquez J Chiarelli P. The Berg Balance Scale has high intra- and inter-rater reliability but absolute reliability varies across the scale: a systematic review. J Physiother. (2013) 59:93–9. 10.1016/S1836-9553(13)70161-9 23663794

[18] Tang YH. Hamilton Depression Rating Scale. Shanghai Arch Psychiatr. (1984) 2:61–4.

[19] Tombaugh TN McIntyre NJ. The mini-mental state examination: a comprehensive review. J Am Geriatr Soc. (1992) 40:922–35. 10.1111/j.1532-5415.1992.tb01992.x 1512391

[20] Li H Jia J Yang Z. Mini-mental state examination in elderly chinese: a population-based normative study. J Alzheimers Dis. (2016) 53:487–96. 10.3233/JAD-160119 27163822

[21] Gan J Wan Y Shi J Zhou M Lou Z Liu Z. A survey of subjective constipation in Parkinson's disease patients in shanghai and literature review. BMC Neurol. (2018) 18:29. 10.1186/s12883-018-1034-3 29544459 PMC5856226

[22] Nieuwboer A Rochester L Herman T Vandenberghe W Emil GE Thomaes T. Reliability of the new freezing of gait questionnaire: agreement between patients with Parkinson's disease and their carers. Gait Posture. (2009) 30:459–63. 10.1016/j.gaitpost.2009.07.108 19660949

[23] Hely MA Reid WG Adena MA Halliday GM Morris JG. The Sydney multicenter study of Parkinson's disease: the inevitability of dementia at 20 years. Mov Disord. (2008) 23:837–44. 10.1002/mds.21956 18307261

[24] Virmani T Moskowitz CB Vonsattel JP Fahn S. Clinicopathological characteristics of freezing of gait in autopsy-confirmed Parkinson's disease. Mov Disord. (2015) 30:1874–84. 10.1002/mds.26346 26234730

[25] Kalia LV Lang AE. Parkinson's disease. Lancet. (2015) 386:896–912. 10.1016/S0140-6736(14)61393-3 25904081

[26] Ehgoetz Martens KA Lukasik EL Georgiades MJ Gilat M Hall JM Walton CC. Predicting the onset of freezing of gait: a longitudinal study. Mov Disord. (2018) 33:128–35. 10.1002/mds.27208 29150872

[27] Selikhova M Williams DR Kempster PA Holton JL Revesz T Lees AJ. A clinico-pathological study of subtypes in Parkinson's disease. Brain. (2009) 132(Pt 11):2947–57. 10.1093/brain/awp234 19759203

[28] Herman T Shema-Shiratzky S Arie L Giladi N Hausdorff JM. Depressive symptoms may increase the risk of the future development of freezing of gait in patients with Parkinson's disease: findings from a 5-year prospective study. Parkinsonism Relat Disord. (2019) 60:98–104. 10.1016/j.parkreldis.2018.09.013 30236826

[29] Nieuwboer A Giladi N. Characterizing freezing of gait in Parkinson's disease: models of an episodic phenomenon. Mov Disord. (2013) 28:1509–19. 10.1002/mds.25683 24132839

[30] Weiss D Schoellmann A Fox MD Bohnen NI Factor SA Nieuwboer A. Freezing of gait: understanding the complexity of an enigmatic phenomenon. Brain. (2020) 143:14–30. 10.1093/brain/awz314 31647540 PMC6938035

[31] Martens KAE Hall JM Gilat M Georgiades MJ Walton CC Lewis SJG. Anxiety is associated with freezing of gait and attentional set-shifting in Parkinson's disease: a new perspective for early intervention. Gait Posture. (2016) 49:431–6. 10.1016/j.gaitpost.2016.07.182 27513741

[32] Ehgoetz Martens KA Hall JM Georgiades MJ Gilat M Walton CC Matar E. The functional network signature of heterogeneity in freezing of gait. Brain. (2018) 141:1145–60. 10.1093/brain/awy019 29444207

[33] Pimenta M Moreira D Nogueira T Silva C Pinto EB Valenca GT. Anxiety independently contributes to severity of freezing of gait in people with parkinson's disease. J Neuropsychiatry Clin Neurosci. (2019) 31:80–5. 10.1176/appi.neuropsych.17090177 30187821

[34] Walton CC Shine JM Hall JM O'Callaghan C Mowszowski L Gilat M. The major impact of freezing of gait on quality of life in Parkinson's disease. J Neurol. (2015) 262:108–15. 10.1007/s00415-014-7524-3 25319020

